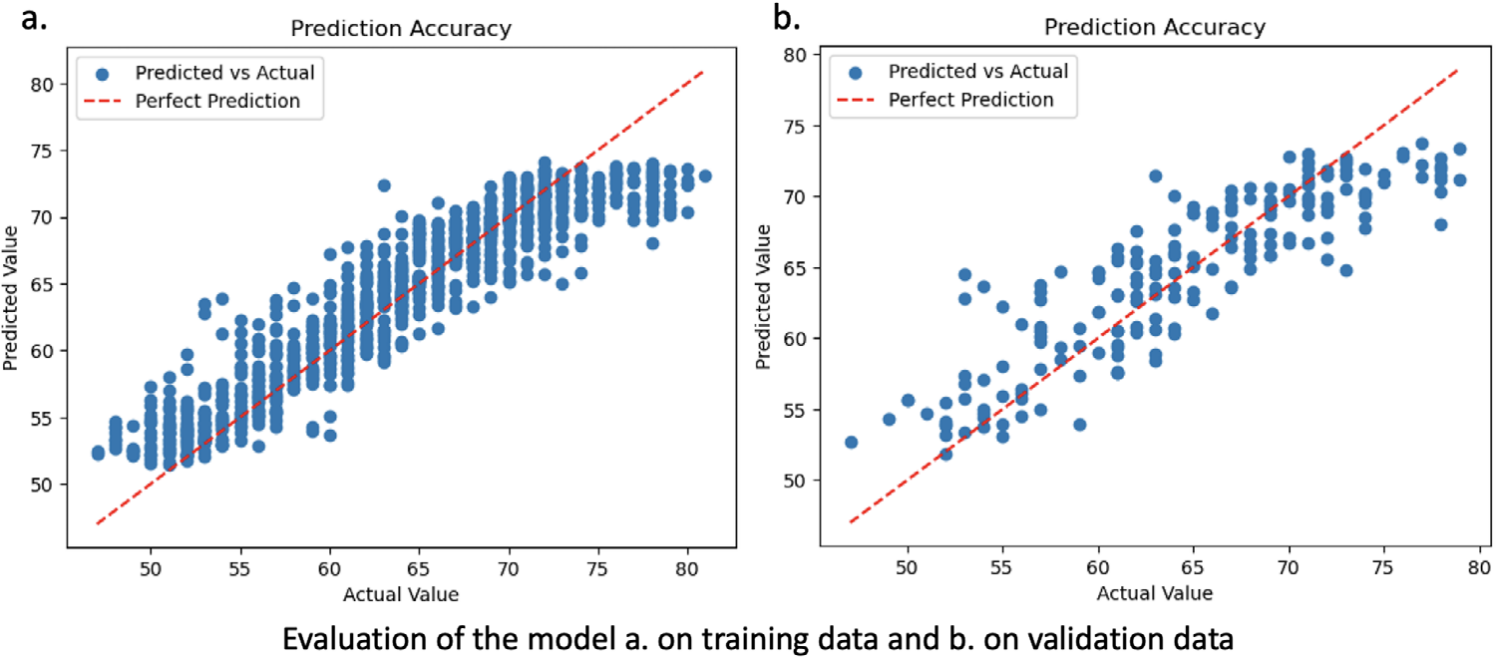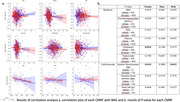# Evaluating the Impact of Cardiometabolic Risk Factors on Neuroimaging‐Based Brain Age: A Deep Learning Approach

**DOI:** 10.1002/alz.095769

**Published:** 2025-01-09

**Authors:** Fatemeh Tavakoli, Da Ma, Yaorong Ge

**Affiliations:** ^1^ University of North Carolina at Charlotte, Charlotte, NC USA; ^2^ Wake Forest University School of Medicine, Winston Salem, NC USA; ^3^ Wake Forest University School of Medicine, Winston‐Salem, NC USA

## Abstract

**Background:**

Mid‐life and late‐life cardiometabolic abnormality are known modifiable risk factors for late‐life dementia. Neuroimaging‐based “brain age” has shown as a surrogate marker for brain health. This study aims to investigate the relationship between effect of cardiometabolic risk factors (CMRF) and accelerated brain aging,

**Method:**

T1‐weighted 3D MRIs from a total of 965 participants (47‐81 years, 53% female) in the UKBiobank data were included in this study. (80%( = 772) training and 20%( = 193) validation). 9 CMRF was extracted, including diastolic blood pressure (dbp), pulse rate (pr), glycated haemoglobin (HbA1c), HDL, glucos, systolic blood pressure (sbp), cholesterol, triglycerides. A brain‐age model pre‐trained using the UKBiobankd data – the Simple Fully Convolutional Network (SFCN) – was fine‐tuned on the brain‐extracted data derived from FreeSurfer (v5.3.0). The brain age gap (BAG) was calculated as the difference between their chronological age and the predicted brain age. Correlation between the CMRF and BAG was derived using the generalized linear model controlling the demographic factors, including age and sex.

**Result:**

The model exhibited high accuracy with a training MAE of 2.05 years and a validation MAE of 2.77 years. Significant associations with the BAG were observed for sbp across genders with a p‐value of 0.0415, with gender‐specific correlations found for cholesterol and sbp in females (p = 0.0242 and 0.0163) and no significant correlations between males’ BAG and any CMRF.

**Conclusion:**

This study discerned the significance of cholesterol and systolic blood pressure as predictors of brain age, emphasizing their potential as markers for brain health in this demographic. Additionally, the observed gender disparities in the association of CMRFs with BAG highlight the necessity for gender‐specific considerations in the assessment and intervention of brain aging.